# Identification of a Prognostic Gene Signature for Chemoresistance Prediction in Lung Adenocarcinoma by Screening Mitochondrial Metabolism Gene Sets

**DOI:** 10.3390/ijms27073065

**Published:** 2026-03-27

**Authors:** Binbin Tan, Jinxu Yang, Xibao Zhao, Shanshan Liu

**Affiliations:** Department of Pharmacology, Shenzhen University Medical School, Shenzhen 518055, China

**Keywords:** lung adenocarcinoma, chemoresistance, mitochondrial metabolism, molecular docking

## Abstract

Chemoresistance is a major challenge in lung adenocarcinoma (LUAD) treatment and is associated with mitochondrial metabolism. Using publicly available LUAD transcriptome data, we established a five-gene prognostic signature (*YWHAZ*, *HSPD1*, *NOTCH3*, *PGK1*, and *PPARG*) for LUAD through differential gene expression profiling, univariate Cox analysis, and machine learning–based feature selection. Patients with LUAD were classified into a high-risk group (HRG) and a low-risk group (LRG) based on their risk scores. Enrichment analysis revealed significant differences between the HRG and LRG in multiple pathways related to metabolism and immunity. The immune microenvironment also differed significantly between the two groups, and the prognostic genes were correlated with infiltrating immune cells. A total of 110 compounds exhibited differential sensitivity across the groups. Molecular docking demonstrated a favorable binding affinity between the prognostic genes and the predicted drugs. Furthermore, *YWHAZ* knockdown significantly suppressed cancer cell proliferation in cell and animal models. In addition, *YWHAZ* knockdown markedly reduced cisplatin resistance by downregulating key regulators of the DNA replication and repair pathway, including *POLA1* and *MCM4*. These results provide insight into the molecular mechanisms underlying chemoresistance and identify putative therapeutic targets for LUAD treatment.

## 1. Introduction

Lung cancer is the leading cause of cancer-related deaths worldwide. Non-small-cell lung cancer (NSCLC) accounts for approximately 85% of all cases [1,2], with lung adenocarcinoma (LUAD) being the most common histological subtype [3]. Notably, the prevalence of LUAD is geographically variable. Among East Asian populations, the incidence of EGFR mutations is approximately 60%, which is considerably higher than the 13–14% observed in European and African populations [4]. In addition, exposure to air pollution may contribute to the development of LUAD, particularly among EGFR-mutant variants [5,6]. Clinically, over half of patients with LUAD are diagnosed at advanced stages (III/IV), which is associated with poor outcomes and reduced survival rates [7]. Treatment for LUAD includes surgical excision, chemotherapy, radiation, molecular targeting, and immune checkpoint blockade. The use of targeted agents against EGFR, ALK, and KRAS, combined with immune checkpoint inhibitors, has yielded substantial improvements in advanced disease outcomes [8,9]; however, a major obstacle in LUAD treatment is the emergence of drug resistance [10]. With respect to EGFR-TKIs, although the initial response is high, resistance develops in most patients within 1–2 years. The T790M mutation is a recognized resistance mechanism; however, nearly half of the cases of resistance lack a molecular explanation [11]. The PD-1/PD-L1 inhibitors, which are immune checkpoint inhibitors, are effective in less than 30% of patients and exhibit high heterogeneity [12,13]. In addition, multidrug resistance is a major concern with chemotherapeutic drug regimens. Therefore, the identification of the underlying molecular mechanisms is required to treat drug resistance in LUAD.

Chemoresistance involves various mechanisms, such as drug efflux, DNA repair, impaired programmed cell death, epithelial–mesenchymal transition (EMT), and alterations in the tumor microenvironment [14]. Of these, mitochondrial metabolic reprogramming is considered an important contributor [15]. As the cell’s energy factory, mitochondria primarily manage energy metabolism through the tricarboxylic acid cycle (TCA) and oxidative phosphorylation (OXPHOS). Unlike normal cells, most cancer cells utilize glycolysis over mitochondrial OXPHOS as an energy source, even in the presence of oxygen, which is known as the Warburg effect [16]. However, in LUAD and other cancers, mitochondrial metabolism is not inactive but undergoes adaptive changes to meet the increased energy and biosynthesis demands, which contribute to chemoresistance. Improved OXPHOS drives ATP-based drug efflux pumps and offers metabolic precursors that promote tumor survival [17]. Mitochondria also play an important role in modulating reactive oxygen species (ROS) and activating antiapoptotic mechanisms, which enhances chemoresistance [18,19]. Thus, mitochondrial metabolism is an important adaptive drug resistance characteristic in LUAD. Because of this reliance on metabolism, there has been increased focus on establishing expression profiles for mitochondrial energy metabolism-related genes (MRGs). MRG expression is associated with clinical outcomes and chemoresistance in LUAD. For example, targeting PDK1 increases osimertinib sensitivity in NSCLC cells [20]. These results suggest that MRGs contribute to chemoresistance in LUAD.

In this study, we systematically analyzed the correlation between drug resistance–related genes and MRGs in LUAD using a combination of bioinformatic analysis and experimental validation. An MRG-based prognostic risk model was established, which exhibited significant predictive power for patient outcomes and provided insight into the underlying mechanisms. Furthermore, we predicted potential therapeutic agents targeting these prognostic genes and determined their binding affinity through molecular docking. The results of this study provide insight into the mechanisms of chemoresistance in LUAD and offer a theoretical foundation for the development of precision therapeutics.

## 2. Results

### 2.1. Discovery of 20 Candidate Genes and Examining Their Biological Roles

**Figure 1:**
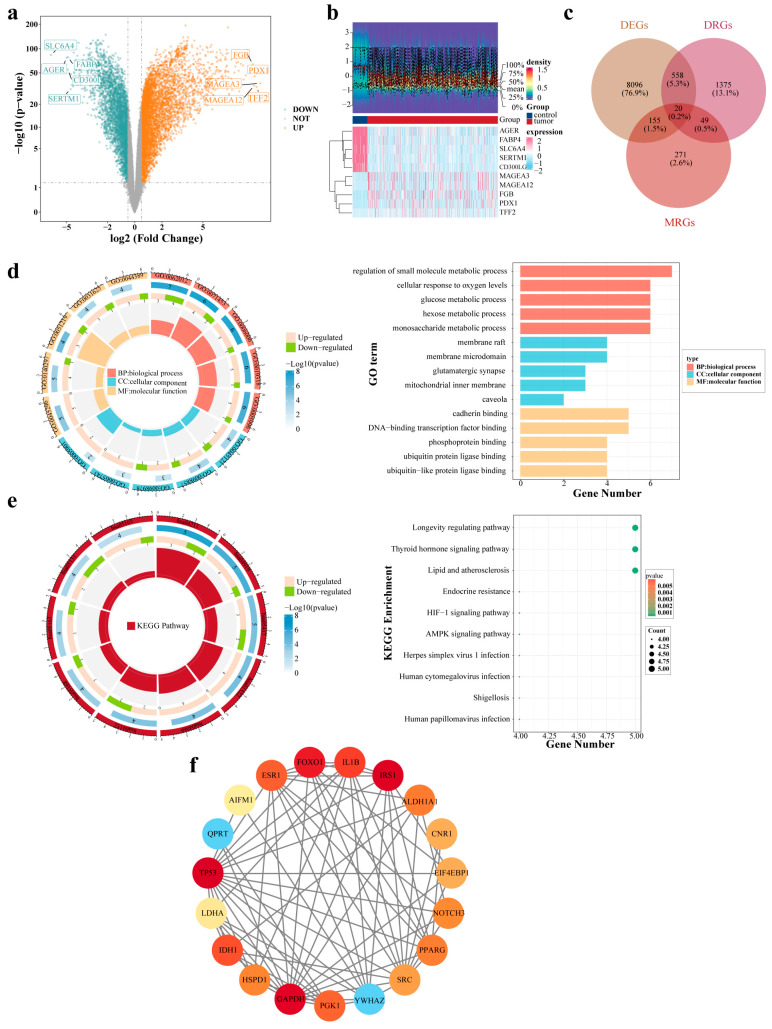
Identification and functional enrichment analysis of candidate genes in LUAD. (**a**) Volcano plot of the DEGs. (**b**) Heatmap of the DEGs. The top portion shows expression density heatmaps for the top five upregulated and downregulated genes with the largest fold-changes and displays lines for 5 quantiles and the mean. The bottom portion is the expression heatmap, in which each column represents a sample and each row indicates the expression level of a gene among the samples. (**c**) Venn diagram of the candidate genes. (**d**) GO enrichment analysis of the candidate genes. (**e**) KEGG enrichment analysis of the candidate genes. (**f**) PPI network of the candidate gene-encoded proteins.

Differential expression analysis was conducted on The Cancer Genome Atlas (TCGA) LUAD dataset (TCGA-LUAD) by comparing tumor versus control cohorts, which yielded 8829 differentially expressed genes (DEGs). Relative to the controls, 3382 genes exhibited reduced expression, whereas 5447 genes showed elevated expression in LUAD specimens. The results were visualized using volcano plots and heatmaps (Figure 1a,b). The intersection of 8829 DEGs with 495 MRGs and 2002 drug resistance–associated genes (DRGs) revealed 20 candidate genes (Figure 1c), which exhibited significant enrichment across 1168 Gene Ontology (GO) terms (*p* < 0.05), encompassing 1029 biological processes (BPs), 26 cellular components (CCs), and 113 molecular functions (MFs; Table S1). The five top enriched BPs included “regulation of small molecule metabolic process” and “cellular response to oxygen levels.” The leading CCs included “membrane raft” and “membrane microdomain.” The predominant MFs were “cadherin binding” and “phosphoprotein binding” (Figure 1d). In addition, Kyoto Encyclopedia of Genes and Genomes (KEGG) analysis revealed the enrichment of 20 candidate genes among 56 pathways (*p* < 0.05) (Table S2), with the top 10 pathways including “longevity regulating pathway” and “thyroid hormone signaling pathway” (Figure 1e). After excluding isolated nodes, a protein–protein interaction (PPI) network incorporating 19 candidate gene-encoded proteins was established, which included TP53 and IRS1 (Figure 1f).

### 2.2. Acquisition of Five Prognostic Genes

**Figure 2:**
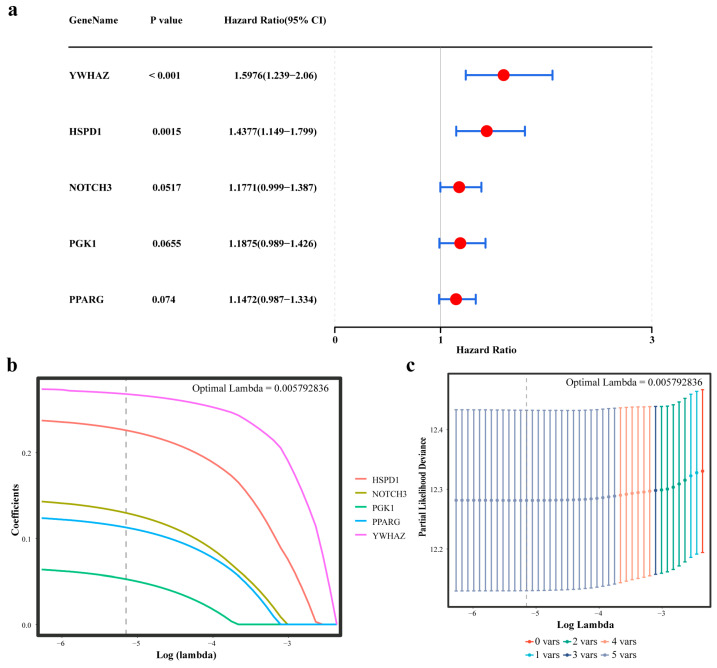
Screening of prognostic genes associated with mitochondrial energy metabolism and chemoresistance in LUAD. (**a**) Univariate Cox regression analysis of the candidate genes. A smaller range of the CI corresponds to higher credibility. (**b**,**c**) LASSO regression analysis. (**b**) The *x*-axis is displayed as log(lambda), and the *y*-axis represents the cross-validation error. (**c**) Different colored dots represent the mean square error and its upper and lower standard deviations. A smaller mean square error indicates a better model. The number at the top indicates the count of independent variables remaining in the model (not necessarily decreasing monotonically). The first dashed line indicates the point of minimum mean square error; the second dashed line marks the position of the simplest model obtainable by allowing one standard deviation from the lowest point.

Within TCGA-LUAD, candidate genes were subject to univariate Cox proportional hazards (PH) regression (*p* < 0.2), followed by PH assumption validation (*p* > 0.05), which yielded the following five candidate prognostic biomarkers: tyrosine 3-monooxygenase/tryptophan 5-monooxygenase activation protein zeta (*YWHAZ*), heat shock protein family D member 1 (*HSPD1*), notch receptor 3 (*NOTCH3*), phosphoglycerate kinase 1 (*PGK1*), and peroxisome proliferator-activated receptor gamma (*PPARG*), which significantly correlated with overall survival (OS) (Figure 2a and Figure S1). Increased *YWHAZ* expression (*p* < 0.001, hazard ratio (HR) [95% confidence interval (CI)] = 1.5976 (1.239–2.06)) and *HSPD1* expression (*p* = 0.0015, HR (95% CI) = 1.4377 (1.149–1.799)) correlated with unfavorable survival outcomes. These five genes were incorporated into Least Absolute Shrinkage and Selection Operator (LASSO) regression modeling, which evaluated the optimal model performance at a lambda value of 0.00579 and retained all five candidate prognostic biomarkers (Figure 2b,c). Therefore, *YWHAZ*, *HSPD1*, *NOTCH3*, *PGK1*, and *PPARG* were designated prognostic biomarkers for LUAD.

### 2.3. Functional Enrichment Analysis of the Five Prognostic Genes

Gene set enrichment analysis (GSEA) revealed that *HSPD1* was enriched in 90 pathways, *NOTCH3* in 89, *PGK1* in 73, *PPARG* in 72, and *YWHAZ* in 74 (Table S3). The top five enriched pathways for these genes are presented in Figure S2. Several pathways were commonly enriched across all five genes, including KEGG_RIBOSOME, KEGG_LEISHMANIA_INFECTION, KEGG_GRAFT_VERSUS_HOST_DISEASE, KEGG_DILATED_CARDIOMYOPATHY, KEGG_TYPE_I_DIABETES_MELLITUS, KEGG_BASAL_CELL_CARCINOMA, and KEGG_DRUG_METABOLISM_CYTOCHROME_P450. These pathways are directly or indirectly associated with mitochondrial function, including energy metabolism, oxidative stress, immune inflammation, cell death, drug metabolism, and disease pathology. For these processes, mitochondria not only act as functional executors but also as disease drivers and therapeutic targets.

### 2.4. The Risk Model Demonstrated Favorable Predictive Performance

**Figure 3:**
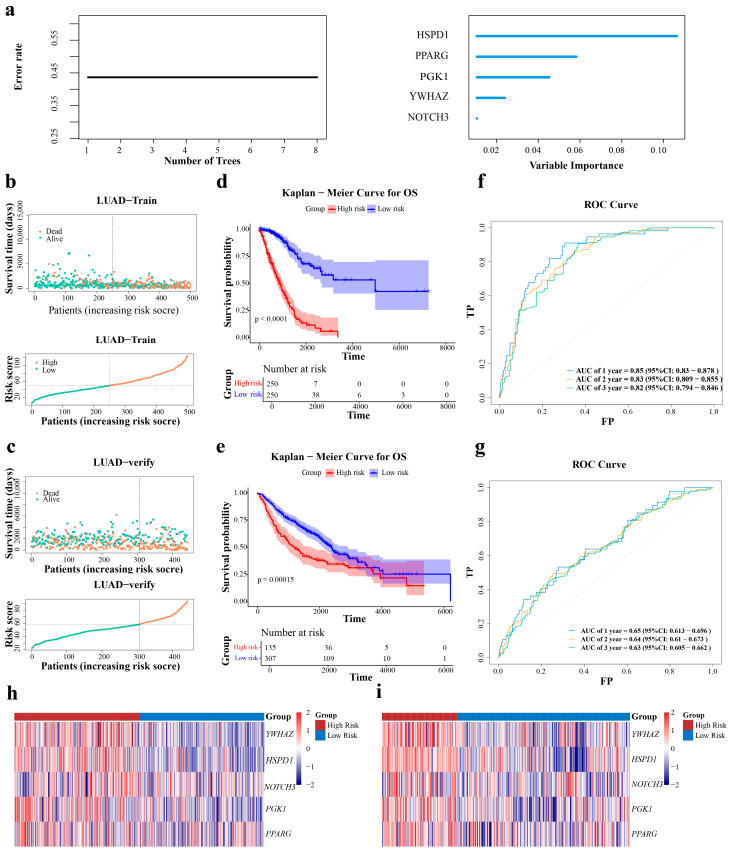
Construction and validation of the prognostic risk model in LUAD. (**a**) Ranking of prognostic genes by importance using random survival forest. (**b**) Risk score curves and survival status distribution (TCGA-LUAD dataset). (**c**) Risk score curves and survival status distribution (GSE68465 dataset). (**d**,**e**) OS Kaplan–Meier survival curves for high- and low-risk groups (TCGA-LUAD and GSE68465). (**f**,**g**) ROC curves for 1-, 2-, and 3-year survival (TCGA-LUAD and GSE68465). (**h**,**i**) Heatmaps for prognostic gene expression in the high- and low-risk groups (TCGA-LUAD and GSE68465).

A risk stratification model was developed within TCGA-LUAD by incorporating *YWHAZ*, *HSPD1*, *NOTCH3*, *PGK1*, and *PPARG*. Of these, *HSPD1* was of maximum importance (Figure 3a). Subsequently, 500 patients with LUAD with comprehensive survival data were stratified into an HRG (n = 250) and an LRG (n = 250) using the optimal risk score cutoff (49.86224). For GSE68465, 442 LUAD tumor specimens were classified into HRG (n = 135) and LRG (n = 307) based on the optimal risk threshold (58.68356). For the TCGA-LUAD and GSE68465 cohorts, increasing risk scores corresponded with a higher probability of mortality (Figure 3b,c). Kaplan–Meier survival analysis revealed significant intergroup differences, with HRG exhibiting a decreased survival probability (TCGA-LUAD: *p* < 0.0001, GSE68465: *p* = 0.00015; Figure 3d,e). Within TCGA-LUAD, the risk model exhibited excellent predictive precision (area under the curve [AUC] > 0.8; Figure 3f). Similarly, GSE68465 had AUC values exceeding 0.6 (Figure 3g). Notably, for both datasets, prognostic gene expression profiles revealed elevated expression in HRG, with *YWHAZ* exhibiting particularly pronounced expression (Figure 3h,i). These results confirmed the robust predictive performance of the risk model for LUAD.

### 2.5. Formation of a Reliable Nomogram Model Based on Prognostic Factors

**Figure 4:**
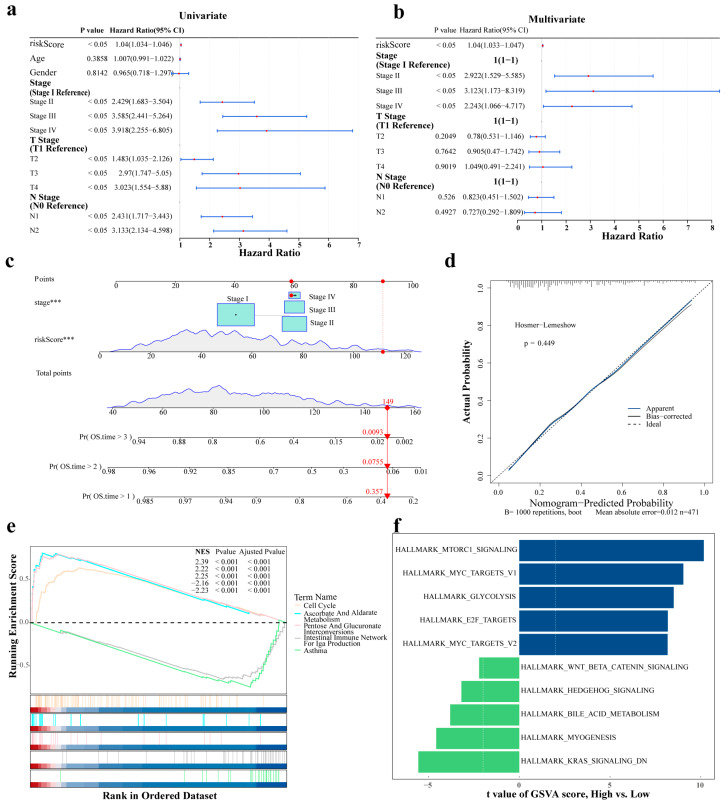
Development of a nomogram and functional pathway analysis based on the risk model. (**a**) Univariate Cox regression analysis of the clinical factors. (**b**) Multivariate Cox regression analysis of the independent prognostic factors. (**c**) Nomogram for predicting 1-, 2-, and 3-year OS probability. Each variable corresponds to a scale-marked line segment reflecting its value range; the length of the segment reflects the contribution of each factor to the outcome. “Points” indicate the single score for each variable value. “Total Points” represents the sum of the individual scores for all variables. *** *p* < 0.001. (**d**) Calibration curve of the nomogram. (**e**) GSEA of the significantly enriched pathways between the HRG and LRG. (**f**) GSVA showing differentially enriched pathways between the HRG and LRG.

Using univariate analysis, PH assumption testing, and multivariate Cox regression analysis, risk score and stage were identified as significant prognostic factors for LUAD (Figure 4a,b and Figure S3; Table 1). Next, a nomogram was established to evaluate the predictive precision of integrating the risk score and stage for estimating survival probability (Figure 4c). When the total score was 149, the corresponding probabilities were as follows: OS time > 1:0.357; OS time > 2:0.0755; OS time > 3:0.0093. These results indicate that an increase in the total risk score and stage was associated with a higher probability of death in patients with LUAD. Additionally, the nomogram model demonstrated a low diagnostic error rate (Hosmer–Lemeshow [HL] test: *p* = 0.449), as evidenced by the calibration curve (Figure 4d). These findings suggest that independent prognostic factors effectively predict LUAD prognosis.

### 2.6. Differences in Enrichment Pathways Between HRG and LRG

The biological pathways involved in HRG and LRG were further analyzed. GSEA identified 60 signaling pathways that were significantly enriched in these groups (|normalized enrichment score [NES]| > 1, false discovery rate [FDR] < 0.25, and *p* < 0.05; Table S4), including “ascorbate and aldarate metabolism” and “asthma” (Figure 4e). Gene set variation analysis (GSVA) revealed that 27 pathways with significant differences between HRG and LRG were collectively enriched (|t| > 2, *p* < 0.05; Table S5). Of these, compared with LRG, “glycolysis” in the HRG was significantly upregulated, whereas “bile acid metabolism” was significantly downregulated (Figure 4f). These results indicate that relevant signaling pathways were significantly enriched between groups, involving metabolism, immunity, and other BPs.

### 2.7. Estimation of the Tumor Immune Microenvironment

**Figure 5:**
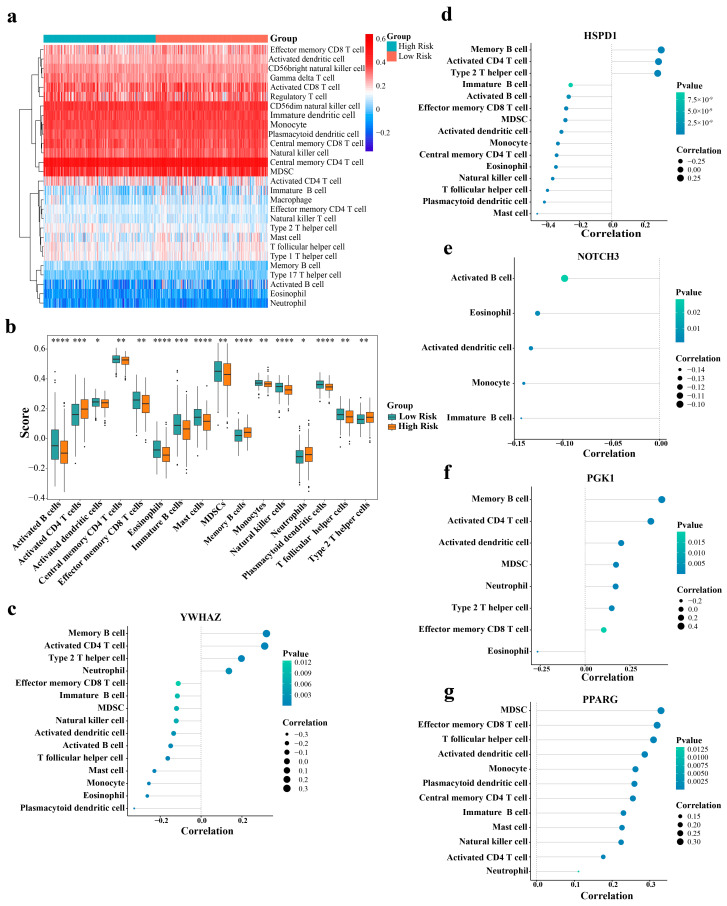
Analysis of the immune cell differences between the high- and low-risk groups. (**a**) Immune cell infiltration profiles for 28 cell types in the HRG and LRG. (**b**) Differential immune cell infiltration between the HRG and LRG. (**c**–**g**) Correlation analysis between prognostic genes (*YWHAZ*, *HSPD1*, *NOTCH3*, *PGK1*, and *PPARG*) and differentially infiltrated immune cells. ns: not significant, * *p* < 0.05, ** *p* < 0.01, *** *p* < 0.001, **** *p* < 0.0001.

We performed comparative immune profiling between high- and low-risk patient groups. Figure 5a illustrates the distribution patterns of 28 distinct immune populations within the HRG and LRG cohorts from the TCGA-LUAD dataset. Notably, central memory CD4 T lymphocytes showed a substantial presence across both risk categories. A statistical comparison identified significant differences in 16 immune cell populations between the two groups (*p* < 0.05), with activated B cells (*p* = 6.2 × 10^−6^) and eosinophils (*p* = 1.2 × 10^−6^) showing pronounced variations (Figure 5b). Next, we examined the correlations between the identified prognostic markers and differentially abundant immune cell populations. *YWHAZ* (cor = 0.311, *p* = 1.06 × 10^−12^) and *PGK1* (cor = 0.367, *p* = 2.403 × 10^−17^) exhibited robust positive associations with activated CD4 T lymphocytes. Conversely, *YWHAZ* (cor = −0.33, *p* = 6.18 × 10^−14^) and *HSPD1* (cor = −0.41, *p* = 3.197 × 10^−22^) showed inverse relationships with plasmacytoid dendritic cells (pDCs; Figure 5c–g). Notably, correlations between the composite risk score and differentially abundant immune cells lacked statistical significance (|cor| < 0.3; Figure S4). Taken together, these results indicate that aberrant immune cellular networks distinguish HRG from LRG. In addition, the data suggest that prognostic markers participate in LUAD pathogenesis through crosstalk with specific immune populations, thus establishing a foundation for elucidating disease mechanisms and designing precision therapeutics.

### 2.8. Mitochondrial Metabolism Pathway Analysis in the High- and Low-Risk Groups

Comparison between the HRG and LRG revealed that the MITOCHONDRIA_PATHWAY and OXIDATIVE_PHOSPHORYLATION scores were significantly higher in the HRG (both *p* < 0.05; Figure S5). All of the prognostic genes and the risk scores exhibited significant positive correlations with both pathways (*p* < 0.05; Figure S6). Notably, *HSPD1* had the highest correlation with OXIDATIVE_PHOSPHORYLATION (cor = 0.36, *p* < 0.001), whereas *PGK1* had the highest correlation with MITOCHONDRIA_PATHWAY (cor = 0.33, *p* < 0.001). These results indicate that the prognostic signature is closely associated with mitochondrial metabolic activity, particularly OXPHOS, and support a role for mitochondrial metabolism in LUAD chemoresistance.

### 2.9. Analysis of Prognostic Genes and Somatic Mutations

**Figure 6:**
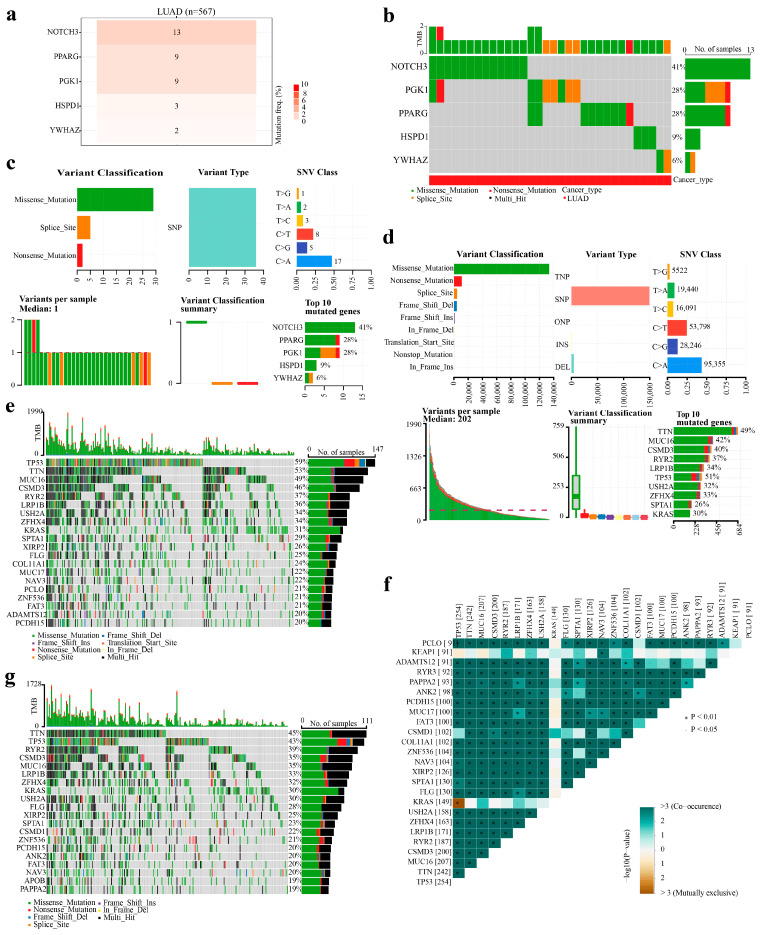
Mutation profiles for the prognostic genes and somatic mutations in LUAD. (**a**) Mutation frequency of the prognostic genes. Gene names are listed on the left side. The central stacked bar chart represents gene mutations; darker colors and higher numbers indicate a larger proportion of samples with mutations. (**b**) Summary of SNV classes for the prognostic genes. Gene names are listed on the left side. The central plot shows SNV classes: green for missense mutations, red for nonsense mutations, and yellow for splice site mutations. (**c**) SNV classification summary for the prognostic genes, showing mutation types and their frequencies. (**d**) Overall mutation landscape. The plots show variant classification, variant type, SNV class, number of variants per sample, and the top 10 mutated genes. (**e**) Mutation distribution in the HRG. (**f**) Mutation distribution in the LRG. (**g**) Interaction analysis of gene mutations between HRG and LRG patients, highlighting co-occurrence or mutual exclusivity.

We analyzed the mutational status of the five prognostic genes in patients with TCGA-LUAD. The results indicated that 13% and 9% of the patients had mutations in *NOTCH3* and PPARG, respectively (Figure 6a). In addition, 41% of *NOTCH3* mutations were missense mutations, whereas 28% of *PGK1* mutations were missense, nonsense, or splice site mutations (Figure 6b,c). Considering the role of genetic alterations in oncogenesis and tumor progression, mutation spectra were derived for patients with LUAD stratified by HRG and LRG from the TCGA database. The results indicated that genes with missense variants were the most frequent in variant categories (Figure 6d). Genes with single-nucleotide polymorphisms were the most common in variant types. Among single-nucleotide variants (SNVs), the “C > T” substitution was observed, whereas the replacement of cytosine (C) with thymine (T) was the most prevalent. In addition, the top 10 genes with the highest mutation frequencies were *TTN*, *MUC16*, *CSMD3*, *RYR2*, *LRP1B*, *TP53*, *USH2A*, *ZFHX4*, *SPTA1*, and *KRAS*. Further analysis of the HRG and LRG revealed that both groups predominantly contained missense mutations and multiple mutations; however, the gene with the highest mutation frequency in HRG was TP53, whereas TTN was most common in LRG (Figure 6e,f). In addition, most mutant genes exhibited co-occurrence (Figure 6g). The results of the mutation analyses revealed the types and frequencies of mutant genes in patients with LUAD, as well as differences in the patterns between the HRG and LRG. Furthermore, most mutant genes showed co-occurrence, providing insights into the molecular characteristics and potential mechanisms underlying this disease.

### 2.10. Chemotherapy Sensitivity Analysis Between the HRG and LRG

**Figure 7:**
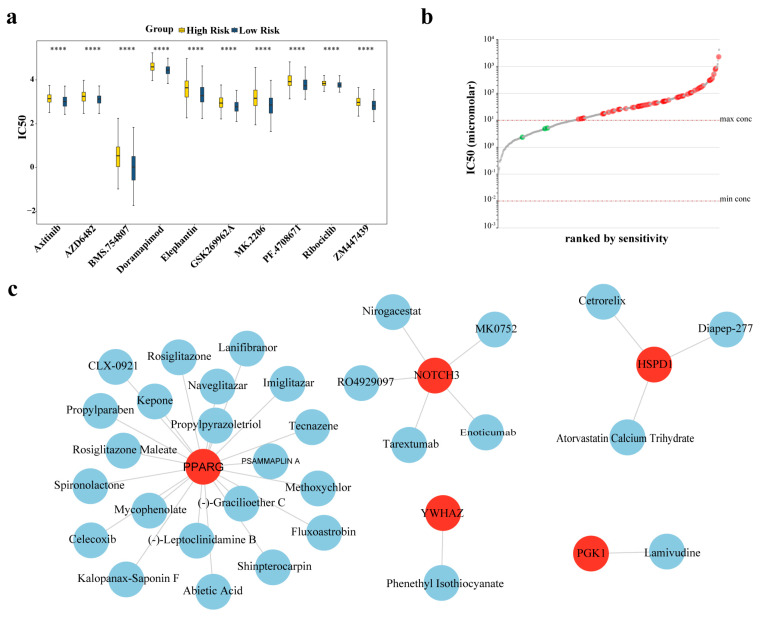
Drug sensitivity analysis and drug prediction for prognostic genes. (**a**) Comparison of IC50 values for LUAD-related drugs between the HRG and LRG. (**b**) Drug-sensitive cell lines identified from the GDSC database. (**c**) Drug–gene interaction network predicted by DGIdb. **** *p* < 0.0001.

Differences in half-maximal inhibitory concentration (IC50) levels for various chemotherapeutic drugs were determined between the HRG and LRG. The IC50 values of 110 chemotherapeutic drugs showed significant differences between the two groups (*p* < 0.05; Table S6). For example, axitinib, AZD6482, and BMS.754807 had higher IC50 values in HRG (Figure 7a). Subsequently, various cell lines (n = 62) sensitive to these drugs were identified (Figure 7b; Table S7), which could provide options for cell line selection in subsequent experiments. These results provide references for the in-depth examination of the pathological mechanisms underlying LUAD.

**Figure 8:**
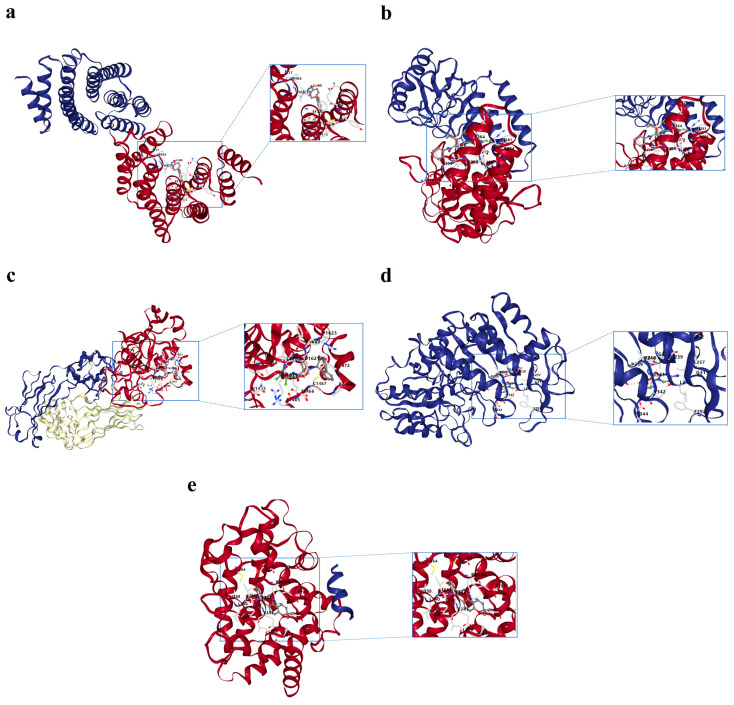
Molecular docking for the prognostic genes. (**a**–**e**) Molecular docking diagrams for drugs with their corresponding prognostic genes: *YWHAZ* (**a**), *HSPD1* (**b**), *NOTCH3* (**c**), *PGK1* (**d**), and *PPARG* (**e**).

Predictions using the Drug–Gene Interaction Database (DGIdb) revealed that five drugs, including enoticumab, could target *NOTCH3*; three drugs, including cetrorelix, could target *HSPD1*; lamivudine could target *PGK1*; and four agents, including protein kinase C, could target *YWHAZ*. In addition, numerous drugs were potential targets for PPARG, with the most relevant top 20 containing celecoxib and tecnazene (Figure 7c). The results of molecular docking indicated that all five prognostic genes had good binding ability with the corresponding drugs (Table 2). Specifically, *YWHAZ* and protein kinase C bind at sites, such as F117 (total score = −7.6; Figure 8a). *HSPD1* and atorvastatin calcium trihydrate had binding sites that included K364 and P368 (total score = −7.8; Figure 8b); *NOTCH3* and tarextumab bound at sites such as C1467 (total score = −8.8; Figure 8c). *PGK1* and lamivudine could potentially bind at sites such as G341 (total score = −6.5; Figure 8d), whereas *PPARG* and shinpterocarpin had binding sites that included K263 and S342 (total score = −6.7; Figure 8e). These results implicated multiple drugs that target these prognostic genes and predicted their binding characteristics. While providing a basis for further exploration, these computational predictions are hypothesis-generating and require confirmation through future in vitro and in vivo functional studies.

### 2.11. YWHAZ Knockdown Inhibits Cancer Cell Proliferation

**Figure 9:**
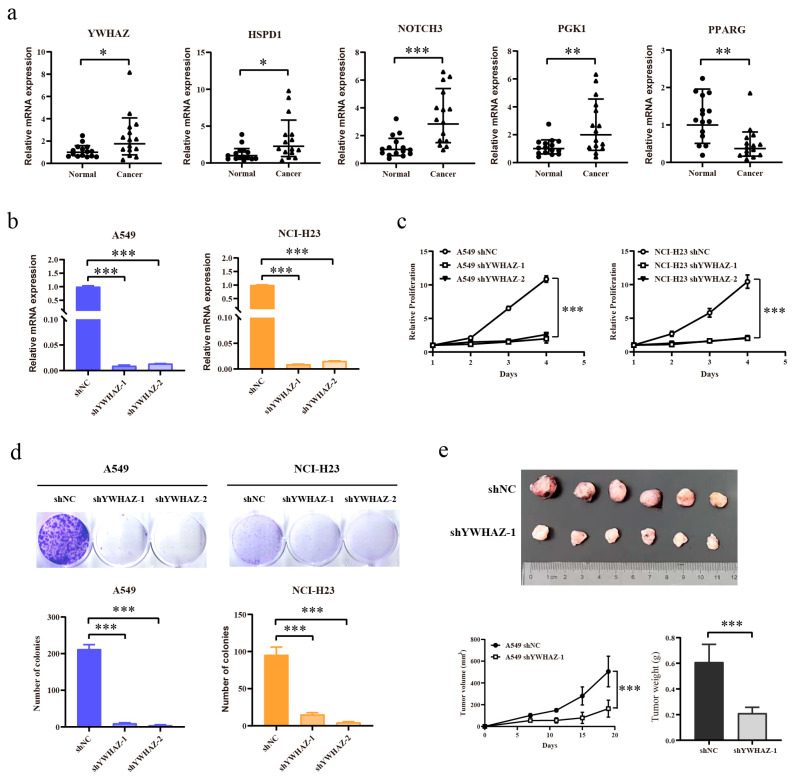
*YWHAZ* knockdown inhibits cancer cell proliferation. (**a**) Expression levels of *YWHAZ*, *HSPD1*, *NOTCH3*, *PGK1*, and *PPARG* mRNAs in LUAD and paired adjacent normal tissues were measured by qRT-PCR. (**b**) A549 and NCI-H23 cells were infected with lentivirus encoding either control shRNA (shNC) or shRNAs targeting YWHAZ (shYWHAZ). Knockdown efficiency was confirmed by qRT-PCR. (**c**) Cell viability of *YWHAZ* knockdown in A549, NCI-H23, and control cells was determined by the CCK-8 assay. (**d**) Colony formation of *YWHAZ* knockdown in A549 and NCI-H23 cells and their parental counterparts. (**e**) Upper panel: images of tumors from *YWHAZ* knockdown A549 (shYWHAZ-1) and control A549 cells (shNC). Lower panel: tumor volume and weight were measured as indicated. * *p* < 0.05, ** *p* < 0.01, *** *p* < 0.001.

We quantified the transcript levels of these five genes using human LUAD cDNA microarrays (Figure 9a). Quantitative RT-PCR revealed that *HSPD1* (*p* < 0.05), *NOTCH3* (*p* < 0.001), *YWHAZ* (*p* < 0.05), and *PGK1* (*p* < 0.01) exhibited significantly increased mRNA levels in malignant tissues relative to corresponding normal tissues, whereas PPARG (*p* < 0.01) exhibited markedly reduced expression in tumor specimens. Functional validation using the CCK-8 viability and clonogenic assays (Figure 9b,c) revealed that genetic silencing of *YWHAZ* markedly suppressed cell proliferation and colony-forming capacity in the A549 and NCI-H23 cell lines (*p* < 0.001). Next, we established a LUAD xenograft model by injecting A549 cells expressing control and *YWHAZ*-targeted shRNA into nude mice (Figure 9d). *YWHAZ* knockdown was associated with reduced tumor volume and weight (*p* < 0.001).

### 2.12. Knockdown of YWHAZ Reduces Resistance to Cisplatin in LUAD Cells

**Figure 10:**
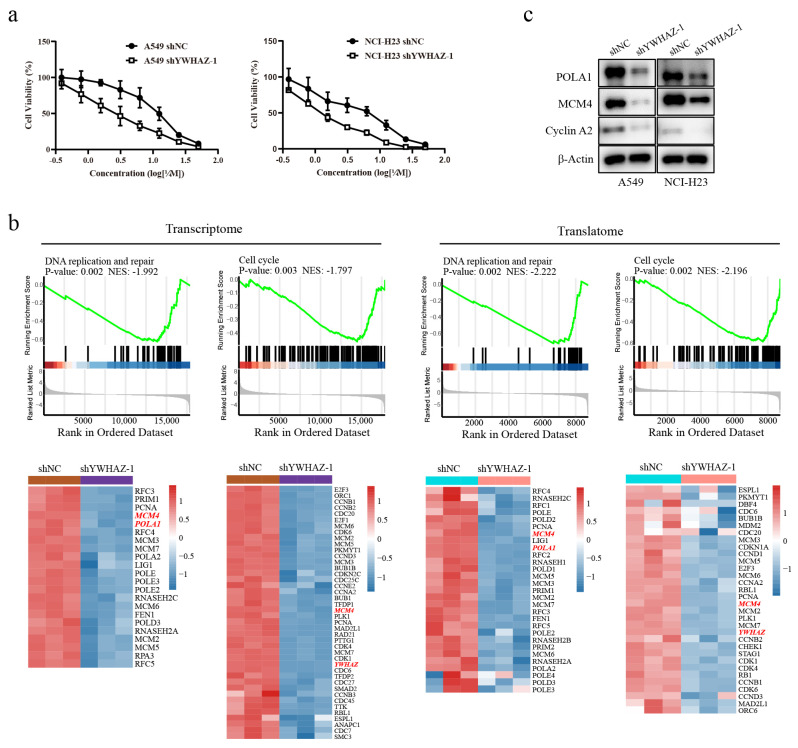
*YWHAZ* knockdown enhances cisplatin sensitivity. (**a**) Cisplatin IC50 for A549 and NCI-H23 cells with or without *YWHAZ* knockdown. (**b**) GSEA of RNA-seq and proteomics data from *YWHAZ* knockdown and control A549 cells. (**c**) The expression of *POLA1*, *MCM4*, and cyclin A2 in *YWHAZ* knockdown and control A549 cells, as determined by immunoblotting.

The CCK-8 assay was used to determine the effects of *YWHAZ* knockdown on cisplatin sensitivity. Cell viability was measured following cisplatin treatment for 48 h (0, 1.56, 3.13, 6.25, 12.5, 25, and 50 μM). *YWHAZ* knockdown significantly enhanced cellular sensitivity to cisplatin (Figure 10a). In A549 cells, the IC50 of cisplatin decreased from 11.2 μM (shNC) to 2.8 μM (shYWHAZ-1). In NCI-H23 cells, the cisplatin IC50 decreased from 5.0 μM (shN.C.) to 1.4 μM (shYWHAZ-1). To identify the underlying mechanism, RNA sequencing (RNA-seq) and proteomics analysis were performed on A549 shYWHAZ-1 and A549 shNC cells. GSEA revealed that genes involved in DNA replication and repair and the cell cycle were markedly downregulated in *YWHAZ* knockdown cells at the transcription and translation levels (Figure 10b). Of these genes, *POLA1* and *MCM4* were the most significantly downregulated genes in the cell cycle and DNA replication and repair pathways. An immunoblotting assay confirmed that *YWHAZ* knockdown inhibited the expression of *MCM4* and *POLA1*. Moreover, cyclin A2 was also downregulated in *YWHAZ* knockdown cells (Figure 10c). These data indicate that knockdown of *YWHAZ* disrupts DNA replication and repair, leading to cell cycle arrest, which in turn sensitizes LUAD cells to cisplatin. *MCM4* and *POLA1* are potential target genes of *YWHAZ*.

## 3. Discussion

Mitochondrial metabolic reprogramming is an important process in chemoresistance. Abnormal manifestation of mitochondrial energy MRGs is closely correlated with LUAD progression and treatment failure. Therefore, we conducted a systematic study on the relationship between genes associated with chemoresistance and MRGs to identify new treatment targets. We identified 20 genes associated with chemoresistance and mitochondrial energy metabolism in LUAD using transcriptomic data available from public databases. An enrichment analysis revealed that these genes are involved in several important pathways, including metabolism, viral infection, and hormone secretion. *YWHAZ*, *HSPD1*, *NOTCH3*, *PGK1*, and *PPARG* were identified as putative prognostic genes, and a highly effective risk model was established. Of these genes, *YWHAZ* was the most promising candidate, and its role in LUAD was validated through in vitro and in vivo experiments.

To determine the biological relevance of these prognostic genes in the etiology and chemoresistance of LUAD, we conducted systematic analyses on the functional role and mechanistic contribution of each gene using the literature and bioinformatic methods. Of the five prognostic genes, *YWHAZ* emerged as a significant candidate. Our results indicated that *YWHAZ* expression was significantly higher in the high-risk subpopulation of LUAD, suggesting an oncogenic role in disease advancement and resistance to treatment. *YWHAZ* is a ubiquitously expressed scaffold protein in eukaryotes that regulates a variety of cellular processes, including signal transduction, cell cycle progression, and stress responses [21,22]. It is highly expressed in various solid tumors, including NSCLC, and is associated with increased growth, evasion of cell death, and enhanced metastasis [23,24,25,26]. *YWHAZ* interacts directly with β-catenin, inhibiting its ubiquitination and promoting its nuclear accumulation, thus activating the EMT process to facilitate metastasis [27]. *YWHAZ* also plays an important role in metabolic reprogramming. Shi et al. demonstrated that silencing *YWHAZ* reduces glycolysis in ovarian cancer cells [28]. Yuan et al. found that circ0515 upregulated *YWHAZ* by binding to miR-328-3p, which resulted in the activation of the PI3K/AKT pathway and increased OXPHOS-succinate metabolism, ultimately inducing cisplatin resistance in LUAD [29]. In the present study, *YWHAZ* overexpression not only affirmed its prognostic value but also provided new insight into LUAD resistance from an energy metabolism perspective. Examining the function of *YWHAZ* in the future may provide new therapeutic targets for LUAD.

*HSPD1*, also known as heat shock protein 60 or Hsp60, is a molecular chaperone that is primarily found in the mitochondrial matrix. It plays an important role in the correct folding and assembly of newly synthesized proteins, particularly enzymes involved in the TCA cycle and OXPHOS [30]. Moreover, *HSPD1* dysregulation disrupts energy metabolism and enhances its function as an innate immune signaling molecule during disease progression [31]. *HSPD1* plays a role in promoting tumor growth, and its high expression has been linked to enhanced cell proliferation, invasion, and poor prognosis in various cancers. For example, blocking *HSPD1* increases ROS levels in colorectal cancer cells, which in turn enhances chemosensitivity [32]. Chandra et al. found that *HSPD1* is associated with proteins involved in apoptosis, such as Apaf-1 and procaspase-3, which enable tumor cells to avoid drug-induced apoptosis [33]. These results are consistent with previous studies identifying *HSPD1* as a poor prognostic marker in NSCLC [34]. Overall, our results confirm a relationship between increased *HSPD1* levels and aggressive LUAD features, which indicates its potential as a prognostic marker and therapeutic target.

In addition to genes directly associated with mitochondrial metabolism, our risk model identified *NOTCH3* as an important receptor in the Notch signaling pathway, highlighting the complex nature of chemoresistance mechanisms in LUAD. *NOTCH3* plays an important role in cell differentiation, proliferation, and tissue homeostasis [35]; however, its deregulation is more widely recognized for contributing to tumor growth, particularly in LUAD [36]. Numerous studies have elucidated the oncogenic roles of *NOTCH3* in LUAD. Xiang et al. reported that *NOTCH3* enhances the invasiveness of LUAD cells by activating tumor-associated mesenchymal cells and promoting collagen production [37]. In addition, *NOTCH3* was upregulated in LUAD samples and chemoresistant cell models, with its genetic silencing suppressing cancer cell proliferation [38]. Dysregulated *NOTCH3* pathway activity correlates with the pathogenesis of various respiratory disorders, such as chronic obstructive pulmonary disease, idiopathic pulmonary fibrosis, and pulmonary hypertension, indicating its role in lung tissue repair [39]. Overall, these results indicate that *NOTCH3* plays an important role in the proliferation, invasiveness, and resistance to therapy in LUAD. Our results provide additional evidence to support these observations.

Metabolic reprogramming through dysregulated glycolytic enzymes represents another important component of treatment resistance in LUAD. We found a significant increase in *PGK1* levels in the high-risk LUAD subgroup, highlighting a role for altered glycolysis in LUAD development. As a key enzyme in glycolysis, *PGK1* converts 1,3-bisphosphoglycerate to 3-phosphoglycerate while producing ATP, thus linking energy production to anabolic pathways [40]. Besides metabolism, *PGK1* acts as an oncogenic protein involved in cancer cell growth, metastasis, blood vessel formation, and chemoresistance, correlating with poor outcomes [41]. Tian found that *PGK1* is overexpressed in NSCLC and was an independent risk factor [42]. GBP1 enhances erlotinib resistance in lung cancer by activating a *PGK1*-mediated EMT pathway [43]. Our results provide further evidence supporting an association between *PGK1* overexpression and LUAD, which suggests its potential as a target for metabolic intervention.

At the level of transcriptional regulation, *PPARG* functions as a primary coordinator, directing the expression of genes involved in energy homeostasis. We identified this gene as a major prognostic marker, which emphasizes the role of transcription factor-mediated metabolic control in LUAD progression and resistance to treatment. PPARG is part of the nuclear hormone receptor superfamily. It acts as a transcription factor activated by ligands and regulates glucose and lipid metabolism, insulin sensitivity, and cell differentiation [44]. The expression and role of PPARG in lung cancer are controversial. In lung squamous cell carcinoma (LSCC), PPARG is not highly expressed, and its activation by treatment regimens can hinder tumor progression [45]; however, the role of PPARG expression in LUAD prognosis remains unclear. PPARG is expressed at relatively high levels in NSCLC adenocarcinoma cell lines, and its activation by ligands causes growth arrest and decreased matrix metalloproteinase activity [46]; however, the prognostic significance of PPARG expression in LUAD remains unclear. Our results provide new insight into its clinical relevance in LUAD and suggest that additional research into the specific functions of PPARG will lead to therapeutic interventions for high-risk patients.

In the present study, pathway enrichment analysis indicated the activation of glycolysis in the high-risk LUAD group, with increased *PGK1* expression highlighting the role of mitochondrial metabolic reprogramming in cancer progression. The Warburg effect provides ATP and biosynthetic precursors for rapid cell growth, while acidifying the tumor microenvironment through lactate production, which promotes immune evasion and angiogenesis [47]. Moreover, glycolytic activation is strongly correlated with resistance to chemotherapy. This pathway contributes to maintaining energy balance within cells and inhibits mitochondrial-mediated apoptosis, thereby enhancing the survival of tumor cells during chemotherapy [48]. In addition, essential enzymes such as *PGK1* can enhance resistance by participating in DNA repair and methylation [49]. Thus, focusing on key glycolytic processes may interfere with both energy production and stress defense systems, presenting a potential therapeutic strategy to overcome resistance and improve outcomes in patients with LUAD.

Besides changes in cellular metabolism, the immune environment within tumors is a key factor in therapeutic resistance. In the present study, the link between our prognostic biomarkers and immune cell distribution patterns was assessed to achieve a more comprehensive view of drug resistance mechanisms. Through detailed immune profiling, we found significant associations between prognostic markers and certain immune populations, indicating their potential role in regulating the immune response during the development of LUAD. We observed a positive association between *YWHAZ* and *PGK1* levels and activated CD4+ T lymphocytes, which suggests that these genes affect the composition of the immune microenvironment. With respect to *PGK1*, this connection may arise from its role in metabolism, because lactate produced during glycolysis leads to T cell dysfunction and fosters an immunosuppressive environment [50]. Conversely, *YWHAZ* and *HSPD1* show opposite associations with pDCs, which are important for initiating antitumor immune responses [51]. Lower pDC infiltration may indicate compromised innate immune surveillance. Such an immune environment encourages tumor immune evasion and promotes LUAD progression and chemoresistance.

Because of the important roles of these prognostic genes in LUAD pathogenesis, we identified potential therapeutic compounds that could target them, thus using molecular docking to predict drug–gene binding affinity. The results indicated strong binding potential between the prognostic genes and specific compounds: *YWHAZ* with protein kinase C, *HSPD1* with atorvastatin calcium trihydrate, *NOTCH3* with tarextumab, *PGK1* with lamivudine, and PPARG with shinpterocarpin. Tarextumab, which is an antibody that targets *NOTCH3*, is effective against pancreatic cancer when used with nab-paclitaxel and gemcitabine [52]. Mujugira et al. reported that lamivudine may be used for HIV-1 and hepatitis B treatment [53], whereas shinpterocarpin exerts therapeutic effects in pancreatic ductal adenocarcinoma because of its high affinity for steroid hormone-responsive proteins [54]. Nevertheless, the mechanisms and effectiveness of these compounds in LUAD require further study.

Our comprehensive analysis provides a list of prognostic genes and their roles in LUAD chemoresistance, although several limitations should be acknowledged. The association between prognostic genes and mitochondrial metabolism was established through a correlation analysis. Future studies will be necessary to assess mitochondrial function by measuring the oxygen consumption rate and extracellular acidification rate using a Seahorse XF Analyzer following *YWHAZ* knockdown, along with the detection of ATP levels and ROS production. The specific mechanism by which *YWHAZ* regulates DNA repair also remains to be determined. Co-immunoprecipitation studies coupled with mass spectrometry to screen for interacting proteins may reveal whether *YWHAZ* attenuates histone modification and downstream DNA repair gene transcription through alterations in metabolic intermediates, such as acetyl-CoA. Furthermore, the small sample size and the breadth of clinical data in public databases may affect how well the model generalizes and predicts. Further studies will focus on validating these results in larger patient cohorts and tracking ongoing investigations of these genes to enhance their clinical application.

## 4. Materials and Methods

### 4.1. Data Acquisition

The TCGA-LUAD dataset was retrieved from the TCGA repository (https://portal.gdc.cancer.gov/ (accessed on 8 April 2025)) and used as the training set. This dataset encompasses 500 tumor specimens with comprehensive survival data from patients with LUAD (tumor cohort), along with 49 adjacent normal tissue specimens (normal cohort). The GSE68465 dataset (platform: GPL96) was obtained from the Gene Expression Omnibus repository (https://www.ncbi.nlm.nih.gov/geo/ (accessed on 8 April 2025)). It comprised 442 LUAD tumor specimens with complete survival records, thus serving as a validation cohort for risk model assessment. Queries were executed using the GeneCards repository (https://www.genecards.org/ (accessed on 8 April 2025)) (cutoff: relevance score >0) and the PubMed platform (https://pubmed.ncbi.nlm.nih.gov/ (accessed on8 April 2025)) with “Mitochondrial Energy Metabolism” as the search term. Following data consolidation and eliminating duplicates, 495 mitochondrial energy MRGs were identified (Table S8). In addition, DRGs were extracted from the DRESIS repository (https://idrblab.org/dresis/ (accessed on 8 April 2025)) and the Molecular Signatures Database (MSigDB; https://www.gsea-msigdb.org/gsea/msigdb (accessed on 8 April 2025)). Post-consolidation and deduplication resulted in 2002 DRGs (Table S9). All datasets were accessed on 8 April 2025.

### 4.2. Discernment and Related Functional Analyses of Candidate Genes

To identify genes associated with chemoresistance and mitochondrial energy metabolism in LUAD, differential expression analysis comparing tumor versus normal cohorts in TCGA-LUAD was done using the DESeq2 package (v 1.38.0) [55] and applying thresholds of *p* < 0.05 and |log_2_ fold change (FC)| > 0.5. Volcano plots showing the DEGs were generated by the ggplot2 package (v 3.4.1) [56]. The top five significantly upregulated or downregulated genes were annotated and arranged by descending log_2_FC magnitude, with corresponding heatmaps created using the ComplexHeatmap package (v 2.14.0) [57]. The overlap among DEGs, MRGs, and DRGs was obtained using the ggvenn package (v 0.1.9) [58], which identified candidate genes associated with mitochondrial metabolism and chemoresistance in LUAD. GO and KEGG pathway enrichment analyses (*p* < 0.05) were performed using clusterProfiler (v 4.2.2) [59] to characterize the biological functions. The top five terms for each GO category (BPs, MFs, and CCs) and top 10 KEGG pathways are displayed based on gene enrichment counts. The STRING repository (https://string-db.org (accessed on 11 April 2025)) was used to establish PPI networks (interaction scores >0.4), with Cytoscape software (v 3.10.2) [60] for the visualization of interactive relationships.

### 4.3. Identification of Prognostic Genes and Construction of a Risk Model

To screen prognostic biomarkers for LUAD, univariate Cox PH regression was performed on the candidate genes in TCGA-LUAD using the survival package (v 3.7.0) [61], which identified genes significantly correlated with LUAD prognosis (*p* < 0.2). The results were visualized using the forestplot package (v 3.1.1) [62]. The cox.zph function assessed PH assumptions for the survival-associated genes (criterion: *p* > 0.05), with the ggcoxzph function used for visualization. Genes satisfying PH assumptions underwent 10-fold cross-validated LASSO regression (family = “cox,” type.measure = “deviance”) using the glmnet package (v 4.1-4) [63]. At optimal lambda values yielding minimal error rates, genes with nonzero coefficients were designated prognostic biomarkers. Subsequently, random survival forest (RSF) modeling, based on the prognostic genes, was implemented using the randomForestSRC package (v 3.2.3) [64] and generated individual patient risk scores. The RSF model was constructed using the following parameters: ntree = 3, mtry = 6, and nodesize = 15, with other parameters set to default values. Specifically, within TCGA-LUAD, bootstrap sampling created B subsets for RSF analysis. Out-of-bag samples facilitated evaluation, and individual survival trees were independently constructed from each subset, with cumulative hazard functions (CHF) calculated. These CHFs were averaged to derive integrated CHF values, with prediction errors determined using OOB-derived integrated CHF. For patient “*x*,” the hazard function at time “*t*” was determined by averaging predictions across all survival trees as follows:

(1) $$h\left(t\left|x\right.\right)=\frac{1}{B}\sum_{i=1}^{B}h_{i}\left(t\left|x\right.\right)$$

where $h_{i}\left(t\left|x\right.\right)$ represents the predicted result of the i-th tree for individual “*x*” at time “*t*.”

The optimal cutoff value for risk score stratification was determined using the surv_cutpoint function from the survminer package (v 0.4.9), which employs maximally selected rank statistics to identify the threshold yielding optimal separation between survival outcomes. Using optimal risk score cutoff values, 500 tumor samples from TCGA-LUAD with complete survival data were stratified into HRG and LRG. The risk curves illustrate risk score variations and survival status distributions between the HRG and LRG. Heatmaps for prognostic gene expression patterns were generated using the ComplexHeatmap package (v 2.14.0). Kaplan–Meier survival curves for the HRG and LRG were generated using the survival package (v 3.7.0), with survival outcome disparities evaluated using the log-rank test (*p* < 0.05). Receiver operating characteristic (ROC) curves for 1-, 2-, and 3-year survival were plotted using the survivalROC package (v 1.0.3.1) [65] and calculating the corresponding AUC values (AUC > 0.6). Finally, risk model validation in GSE68465 was done using methodologies consistent with TCGA-LUAD analysis to evaluate generalizability.

### 4.4. Nomogram Model Construction and Assessment

Univariate Cox regression (HR ≠ 1, *p* < 0.05) was used to initially select prognostic variables, followed by PH assumption testing (*p* > 0.05). Multivariate Cox regression identified final independent prognostic determinants, with results visualized using the forestplot package (v 3.1.1). Based on these determinants, the rms package (v 6.5.0) [66] constructed a nomogram predicting 1-, 2-, and 3-year survival probabilities for patients with LUAD. Within the nomogram, each independent factor received unique point assignments, with higher cumulative points corresponding to reduced survival rates. Finally, calibration curves assessing nomogram accuracy were plotted using the rms package (v 6.5.0), and reliability was evaluated using the HL test (*p* > 0.05).

### 4.5. Gene Set Enrichment Analysis and Gene Set Variation Analysis

GSEA was done to identify the biological functions of prognostic genes during LUAD progression. Differential gene expression between HRG and LRG was carried out using the DESeq2 package (v 1.38.0), with log_2_FC values ranked in descending order. The reference gene set “c2.cp.kegg.v7.5.1.entrez.gmt” from MSigDB was used. GSEA was performed using the clusterProfiler package (v 4.2.2) with the following criteria: |NES| >1, FDR < 0.25, and *p* < 0.05. The top five pathways were visualized using *p*-values. To further identify the roles of the prognostic genes, GSVA was performed. GSVA scores for the HRG and LRG were analyzed using the GSVA package (v 1.46.0) [67], with score comparisons conducted using the limma package (v 3.58.1) [68] (|t| > 2, *p* < 0.05). The ggplot2 package (v 3.4.1) was used to visualize the top five upregulated and downregulated pathways.

### 4.6. Mitochondrial Metabolism Pathway Analysis

To examine the relationship between the prognostic signature and mitochondrial metabolism, two gene sets were downloaded from MSigDB: BIOCARTA_MITOCHONDRIA_PATHWAY and HALL-MARK_OXIDATIVE_PHOSPHORYLATION. Single-sample GSEA (ssGSEA) was conducted using the GSVA package (v 1.48.2) to calculate enrichment scores for these pathways in each TCGA-LUAD sample. Differences in the enrichment scores between the HRG and LRG were determined using the Wilcoxon test. A Spearman correlation analysis was performed using the psych package (v 2.5.6) to determine the association between prognostic genes, risk scores, and enrichment scores of two mitochondrial pathways. Correlations with *p* < 0.05 were considered statistically significant. For functional enrichment analysis of individual prognostic genes, Spearman correlations were calculated between each core gene and all other genes in the TCGA-LUAD training set. Genes with significant correlations (*p* < 0.05) were ranked by correlation coefficients and then subjected to GSEA using the KEGG gene set “c2.cp.kegg.v7.5.1.symbols.gmt.” Enriched pathways with |NES| > 1 and *p* < 0.05 were retained, and the top five pathways for each gene are presented.

### 4.7. Immune Microenvironment Analysis

Immune cell infiltration levels correlate with LUAD progression. The ssGSEA algorithm was applied to the TCGA-LUAD tumor specimens using the GSVA package (v 1.46.0) to calculate enrichment scores for 28 immune cell types [69] between the HRG and LRG, with heatmap visualization. Wilcoxon tests were used to compare immune cell infiltration differences between the HRG and LRG (*p* < 0.05). Immune cells exhibiting significant infiltration differences were designated differentially infiltrated immune cells and visualized using the ggplot2 package (v 3.4.1). Subsequently, Spearman correlation analyses between prognostic genes and differentially infiltrated immune cells were conducted using the psych package (v 2.2.9) [70] (|correlation coefficient| > 0.3, *p* < 0.05), with the corrplot package (v 0.92) [71] for visualization.

### 4.8. Analyses of Prognostic Gene and Somatic Mutations

To establish mutation profiles for the prognostic genes, including mutation susceptibility types and categories, the genes were queried in the Gene Set Cancer Analysis repository (http://guolab.wchscu.cn/GSCA/#/ (accessed on 11 April 2025)). The “Mutation” module analyzed “SNV summary” and “CNV summary” perspectives to evaluate mutation frequency, single-nucleotide variant (SNV) types, and copy number variation (CNV) patterns. Next, the somatic mutation status was determined. Overall, mutation spectra were plotted by calculating the mutation frequency and exon length for each patient in TCGA-LUAD. To identify somatic mutation differences between the HRG and LRG, the maftools package (v 2.14.0) [72] was used to analyze mutation landscapes and calculate tumor mutation burden values. The top 20 genes were displayed by mutation frequency. The somatic interaction function was used to conduct paired Fisher’s exact tests to analyze co-occurrence and mutational exclusivity among the mutated genes (*p* < 0.05), with the top 25 genes ranked by ascending *p*-values.

### 4.9. Drug Sensitivity Analysis

Chemotherapy represents a widely adopted clinical approach for malignancy treatment, using drugs with low IC50 to suppress cell proliferation. To assess chemosensitivity differences between the HRG and LRG, IC50 values for LUAD-relevant drugs were obtained from the Genomics of Drug Sensitivity in Cancer (GDSC) repository (https://www.cancerrxgene.org/ (accessed on 11 April 2025)). For TCGA-LUAD tumor samples with survival data, drug sensitivity predictions used the oncoPredict package (v 1.2) [73]. The GDSC2 dataset, including RMA-normalized and log_2_-transformed gene expression matrices (GDSC2_expr) and corresponding drug sensitivity data (GDSC2_res, characterized by IC50 values), was used as the training set. For the TCGA-LUAD samples, FPKM expression values were log_2_-transformed and converted to numerical matrices using the lc.tableToNum() function. Only samples matched to the HRG and LRG were retained (train_data). To correct batch effects between the training (cell lines) and test sets (clinical samples), the Empirical Bayes (eb) method was used to eliminate systematic errors arising from different data sources. Wilcoxon tests (*p* < 0.05) were used to compare sensitivity differences between the HRG and LRG. The top 10 drugs exhibiting the most significant differences (ascending *p*-values) were selected for boxplot visualization. Finally, within the GDSC database, the “LUAD” search scope was used to explore the most sensitive cell lines for all significantly different drugs (AUC > 0.7).

### 4.10. Drug Prediction and Molecular Docking

To identify therapeutic compounds targeting prognostic genes, the DGIdb (https://dgidb.org (accessed on 22 April 2025)) was searched for potential drug–gene interactions. Cytoscape software (v 3.10.2) generated drug–prognostic gene interaction networks. Three-dimensional (3D) drug conformations were obtained from the PubChem database (https://pubchem.ncbi.nlm.nih.gov/ (accessed on 11 April 2025)), whereas 3D crystallographic structures of prognostic gene-encoded proteins were acquired from the Protein Data Bank database (https://www.rcsb.org/ (accessed on 11 April 2025)). The CBDock2 server (https://cadd.labshare.cn/cb-dock2/php/index.php (accessed on 11 April 2025)) conducted a molecular docking analysis for drugs showing the strongest interaction scores with their respective prognostic genes. The specific molecular docking parameters are listed in Table S10. The binding energy values reflect ligand-receptor affinity: |total score| > 4.0 indicates detectable binding capacity; |total score| > 5.0 represents good binding capacity; and |total score| > 7.0 signifies robust binding capacity.

### 4.11. Quantitative Real-Time Polymerase Chain Reaction

Human LUAD cDNA microarrays (Cat# MecDNA-HLugA030PG01) containing 15 matched cancer samples were obtained from Shanghai Outdo Biotech (Shanghai, China). Quantitative real-time polymerase chain reaction (qRT-PCR) was performed with FastStart Universal SYBR Green Master Mix (Rox) (Roche, New York, NY, USA) using a 7500 Real-Time PCR System (Applied Biosystems, Carlsbad, CA, USA). Primer sequences are listed in Table S11.

### 4.12. Cell Culture

HEK293T, A549, and NCI-H23 cells were kindly provided by the Cell Bank, Chinese Academy of Sciences (Shanghai, China). A549 cells were cultured in Ham’s F12 (Thermo Fisher Scientific, Waltham, MA, USA) supplemented with 10% fetal bovine serum (FBS; Thermo Fisher Scientific, Waltham, MA, USA). NCI-H23 cells were cultured in RPMI 1640 (Thermo Fisher Scientific, Waltham, MA, USA) supplemented with 10% FBS. HEK293T cells were cultured in Dulbecco’s modified Eagle medium (Thermo Fisher Scientific, Waltham, MA, USA) supplemented with 10% FBS. All cells were maintained at 37 °C in a 5% CO_2_ humidified atmosphere.

### 4.13. Construction of YWHAZ Knockdown Cell Lines

Two independent shRNAs targeting *YWHAZ* (shYWHAZ-1 and shYWHAZ-2) and a negative control shRNA (shNC) were subcloned into the pLKO.1-puro lentiviral vector (Sigma-Aldrich, St. Louis, MO, USA). The targeting sequences are listed in Table S11. For lentivirus packaging, HEK293T cells were co-transfected with the shRNA-containing pLKO.1-puro plasmid, along with the psPAX2 and pMD2.G packaging plasmids using the jetPEI DNA Transfection Reagent (Polyplus, Illkirch, France) based on the manufacturer’s instructions. After 24 h of transfection, the medium was replaced with fresh culture medium. Viral supernatants were then collected at 48 and 72 h post-transfection, filtered through a 0.45-μm filter, and either used immediately or stored at −80 °C.

A549 and NCI-H23 cells were seeded into 6-well plates and infected with lentiviral supernatants in the presence of 8 μg/mL polybrene. After 24 h, the medium was replaced with fresh culture medium containing 5 μg/mL puromycin to select stably transduced cells. Knockdown efficiency was confirmed by qRT-PCR. The primer sequences used for qRT-PCR validation of *YWHAZ* knockdown are listed in Table S11.

### 4.14. Cell Proliferation Assay

For the CCK-8 experiments, cells were seeded into 96-well plates at a density of 2000 cells/well. Cell viability was assessed by adding 10 µL of CCK-8 (Beyotime, Shanghai, China) to 100 µL of culture medium at the specified intervals. The cells were incubated at 37 °C for 1.5 h, followed by absorbance detection at 450 nm using a microplate reader (BioTek, Winooski, VT, USA). Relative proliferation was determined by normalizing to the initial day absorbance. For colony formation assays, the cells were seeded into six-well plates at 1500 cells/well and maintained at 37 °C for 10 days. The medium was refreshed every 3 days. Following incubation, the cells were washed with PBS, fixed with 4% paraformaldehyde, and stained with 0.1% crystal violet. Colonies harboring ≥50 cells were counted under a microscope, and colony formation efficiency was determined.

### 4.15. Experimental Model Animals and Animal Care

BALB/c nude mice (4–5 weeks old; GemPharmatech Corporation, Nanjing, China) were housed in specific pathogen-free conditions at the Shenzhen University Laboratory Animal Centre. A549 cells were transduced with control shRNA (shN.C.) or *YWHAZ*-targeting shRNA-1 (shYWHAZ-1) (5 × 10^6^ cells in 100 μL PBS) and subcutaneously implanted into the right flank of each mouse. Tumor growth was monitored weekly with calipers to track progression. Tumor volume was determined by the following formula: Volume = (length × width^2^)/2. All experimental procedures were conducted in accordance with protocols approved by the Animal Ethics and Welfare Committee of Shenzhen University (2021006).

### 4.16. Statistical Analysis

Statistical evaluations were performed using R software (v.4.2.2). Wilcoxon tests were conducted for intergroup comparative analyses. Two-tailed Student’s *t*-tests and two-way analysis of variance were used as warranted. *p* < 0.05 was considered statistically significant. *p*-value notations: * *p* < 0.05, ** *p* < 0.01, *** *p* < 0.001, ns = not significant.

## 5. Conclusions

In this study, we identified five mitochondrial MRGs (*YWHAZ*, *HSPD1*, *NOTCH3*, *PGK1*, and *PPARG*) as prognostic markers in LUAD and constructed a robust risk model that demonstrated strong predictive performance. These genes are closely associated with chemoresistance and are involved in key processes, including metabolic reprogramming, immune microenvironment modulation, and signaling pathway dysregulation. The model highlights glycolysis activation and specific immune cell infiltration patterns as hallmarks of high-risk disease. Furthermore, molecular docking analysis revealed the binding potential of several compounds to these prognostic genes. It should be emphasized that these computational predictions are solely intended for hypothesis generation, and their binding affinities as well as potential therapeutic value require further validation in future in vitro and in vivo functional studies.

## Figures and Tables

**Table 1:** Assessment of PH assumptions for independent prognostic factors in LUAD.

	Chisq	df	*p* Values
RiskScore	0.718139531114634	1	0.396754855533997
Stage	2.90511996268253	3	0.406486328699415

**Table 2:** Molecular docking for predicted drug–gene interactions targeting prognostic genes.

Gene	Drug	Total Score
*HSPD1*	Atorvastatin calcium trihydrate	−7.8
*NOTCH3*	Tarextumab	−8.8
*PGK1*	Lamivudine	−6.5
*PPARG*	Shinpterocarpin	−6.7
*YWHAZ*	Protein kinase C	−7.6

## Data Availability

The datasets analyzed during the current study are available in the Gene Expression Omnibus (GEO) database repository (http://www.ncbi.nlm.nih.gov/geo/ (accessed on 8 April 2025)) and The Cancer Genome Atlas (TCGA) database (https://portal.gdc.cancer.gov/ (accessed on 8 April 2025)).

## Supplementary Materials

The following supporting information can be downloaded at: https://www.mdpi.com/article/10.3390/ijms27073065/s1.

## Abbreviations

The following abbreviations are used in this manuscript:

LUAD	Lung adenocarcinoma
TCGA	The Cancer Genome Atlas
GEO	Gene Expression Omnibus
GO	Gene Ontology
KEGG	Kyoto Encyclopedia of Genes and Genomes
ROS	Reactive oxygen species
LASSO	Least absolute shrinkage and selection operator
qRT-PCR	Quantitative real-time polymerase chain reaction
IHC	Immunohistochemistry
TMB	Tumor mutation burden
DEGs	Different expression genes
DRGs	Drug resistance-associated genes
MRGs	Mitochondrial energy metabolism-related genes
GSEA	Gene set enrichment analysis
GSVA	Gene set variation analysis
GSCA	Gene set cancer analysis
HRG	High-risk group
LRG	Low-risk group
TCA	Tricarboxylic acid
SNPs	Single nucleotide polymorphisms
DGIdb	Drug-gene interaction database
GDSC	Genomics of drug sensitivity in cancer
IC50	Half-maximal inhibitory concentration
SNVs	Single-nucleotide variants
CNV	Copy number variation
RSF	Random survival forest
CHF	Cumulative hazard functions
HL	Hosmer–Lemeshow
PPI	Protein–protein interaction
BP	Biological processes
CC	Cellular components
MF	Molecular functions
HR	Hazard ratio
CI	Confidence interval
PH	Proportional hazards
OS	Overall survival
AUC	Area under the curve
ROC	Receiver operating characteristic
NES	Normalized enrichment score
FDR	False discovery rate
PDCs	Plasmacytoid dendritic cells
ssGSEA	Single-sample gene set enrichment analysis
PDB	Protein Data Bank
