# Supplementary material for: Identification of a Prognostic Gene Signature for Chemoresistance Prediction in Lung Adenocarcinoma by Screening Mitochondrial Metabolism Gene Sets
Source: Int J Mol Sci. 2026 Mar 27;27(7):3065. doi: 10.3390/ijms27073065 (PMC13073725; doi:10.3390/ijms27073065)
Supplement: Supplementary file 1 [file ijms-27-03065-s001.zip › legends.pdf]

Figure S1: PH assumption testing for the five prognostic genes (YWHAZ, HSPD1, NOTCH3, PGK1, PPARG).

Figure S2: GSEA enrichment analysis results of core genes. (A) HSPD1; (B) NOTCH3; (C) PGK1; (D) PPARG ;(E) YWHAZ; Note: In the upper Enrichment Score line plot, the x-axis represents the ranked genes and the y-axis represents the corresponding Running ES. The peak value in the line plot indicates the Enrichment Score of the gene set for this pathway; the middle section shows the positions of genes within the gene set; the lower part displays the correlation trend of the gene ranking list.

Figure S3: PH assumption testing for independent prognostic factors.

Figure S4: Correlation analysis between the composite risk score and differentially infiltrated immune cells.

Figure S5: Differences in risk scores between groups. Green represents the low-risk group, and orange represents the high-risk group.

Figure S6: Heatmap of correlation analysis between biomarkers and differential immune cells. (A) HSPD1; (B) NOTCH3; (C) PGK1; (D) PPARG ;(E) YWHAZ; (E) risk score; The circle color changing from cyan to blue indicates increasing correlation between genes, risk score and MITOCHONDRIA_PATHWAY and OXIDATIVE_PHOSPHORYLATION. Larger circle size represents stronger correlation. Circles located to the right of the vertical line indicate positive correlation, while those to the left indicate negative correlation;

Table S1: Top enriched GO terms for the 20 candidate genes.

Table S2: Top enriched KEGG pathways for the 20 candidate genes.

Table S3: GSEA enrichment pathways of 5 prognostic genes.

Table S4: Top enriched pathways identified by GSEA between HRG and LRG.

Table S5: Top significantly up- and down-regulated pathways identified by GSVA between HRG and LRG.

Table S6: Chemotherapeutic drugs with significantly different IC50 values between HRG and LRG.

Table S7: LUAD cell lines most sensitive to the significantly different drugs.

Table S8: List of MRGs.

Table S9: List of DRGs.

Table S10: Detailed molecular docking parameters for all target proteins.

Table S11: The primers and shRNA sequence used in this study.
