# Supplementary material for: Identification of a Prognostic Gene Signature for Chemoresistance Prediction in Lung Adenocarcinoma by Screening Mitochondrial Metabolism Gene Sets
Source: Int J Mol Sci. 2026 Mar 27;27(7):3065. doi: 10.3390/ijms27073065 (PMC13073725; doi:10.3390/ijms27073065)
Supplement: Supplementary file 1 [file ijms-27-03065-s001.zip › Table S11.pdf]

Table S11. The primers and shRNA sequence used in this study

Genes	Sequences
HSPD1	Forword: GTGTAGACCTTTTAGCCGATGC
	Reverse: GTGCCAGTACAGTAGCAGTGG
NOTCH3	Forword: CGTGGCTTCTTTCTACTGTGC
	Reverse: CGTTCACCGGATTTGTGTCAC
PPARG	Forword: GCAGGTGATCAAGAAGACGGAGAC
	Reverse: AAATGTTGGCAGTGGCTCAGGAC
PGK1	Forword: GAACAAGGTTAAAGCCGAGCC
	Reverse: GTGGCAGATTGACTCCTACCA
YWHAZ	Forword: TGTAGGAGCCCGTAGGTCATC
	Reverse: GTGAAGCATTGGGGATCAAGA
GAPDH	Forword: TGTGGGCATCAATGGATTTGG
	Reverse: ACACCATGTATTCCGGGTCAAT
shN.C.	CCTAAGGTTAAGTCGCCCTCG
shYWHAZ-1	GCAGAGAGCAAAGTCTTCTAT
shYWHAZ-2	GCAATTACTGAGAGACAACTT
