# Supplementary figures and images for: Identification of a Prognostic Gene Signature for Chemoresistance Prediction in Lung Adenocarcinoma by Screening Mitochondrial Metabolism Gene Sets
Source: Int J Mol Sci. 2026 Mar 27;27(7):3065. doi: 10.3390/ijms27073065 (PMC13073725; doi:10.3390/ijms27073065)

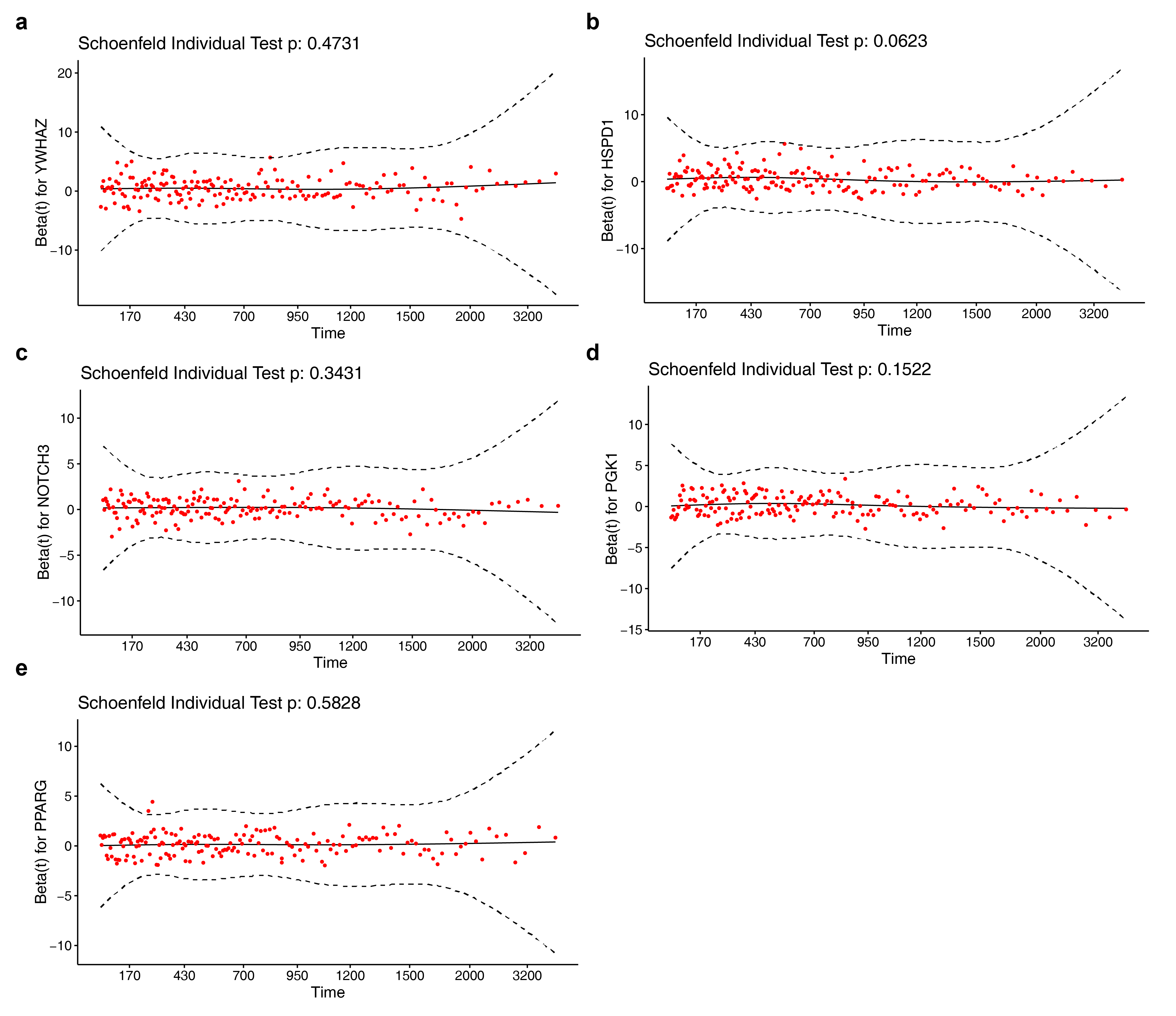

Supplement: Supplementary file 1 [file ijms-27-03065-s001.zip › Figure S1.tif]

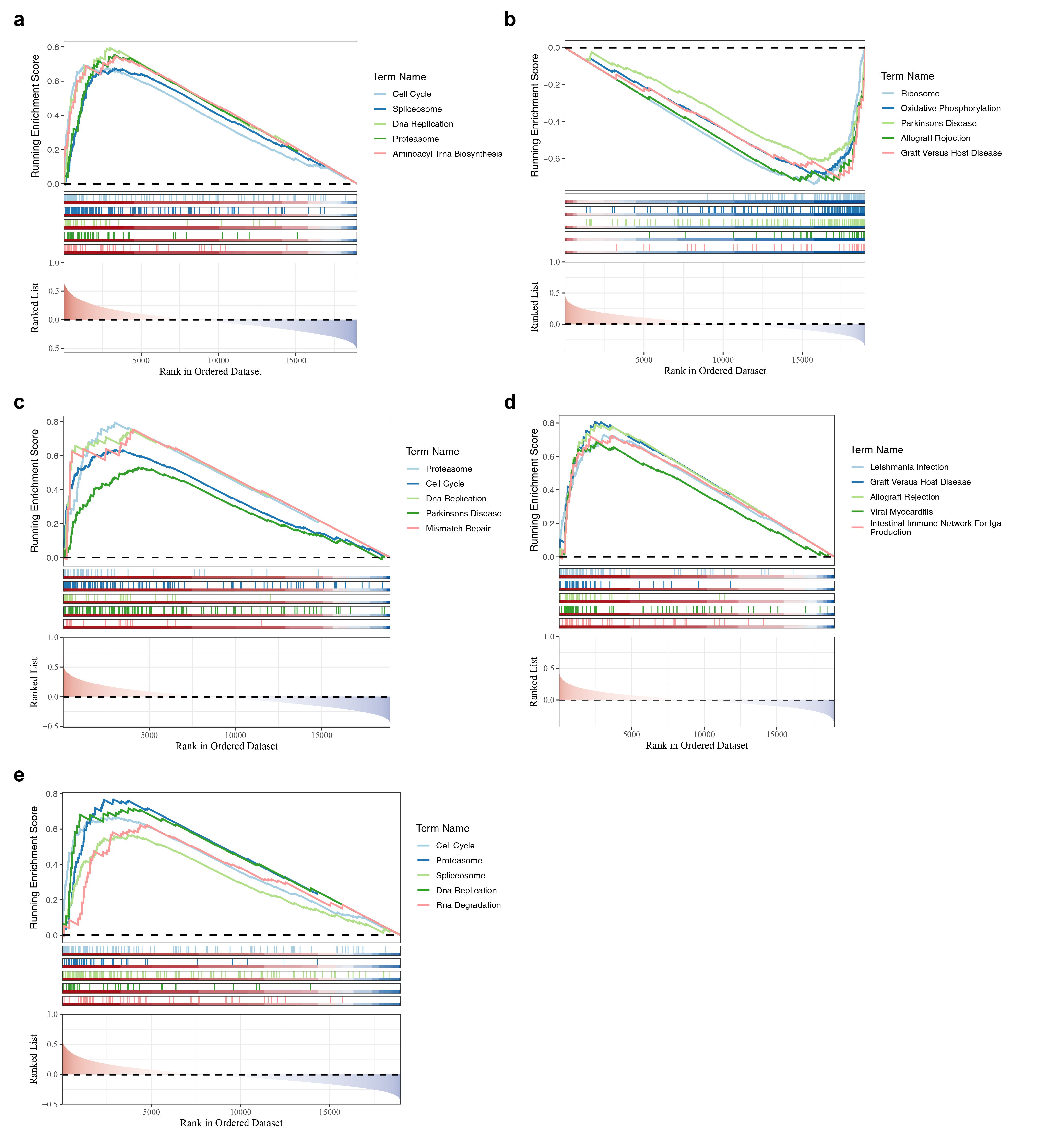

Supplement: Supplementary file 1 [file ijms-27-03065-s001.zip › Figure S2.tif]

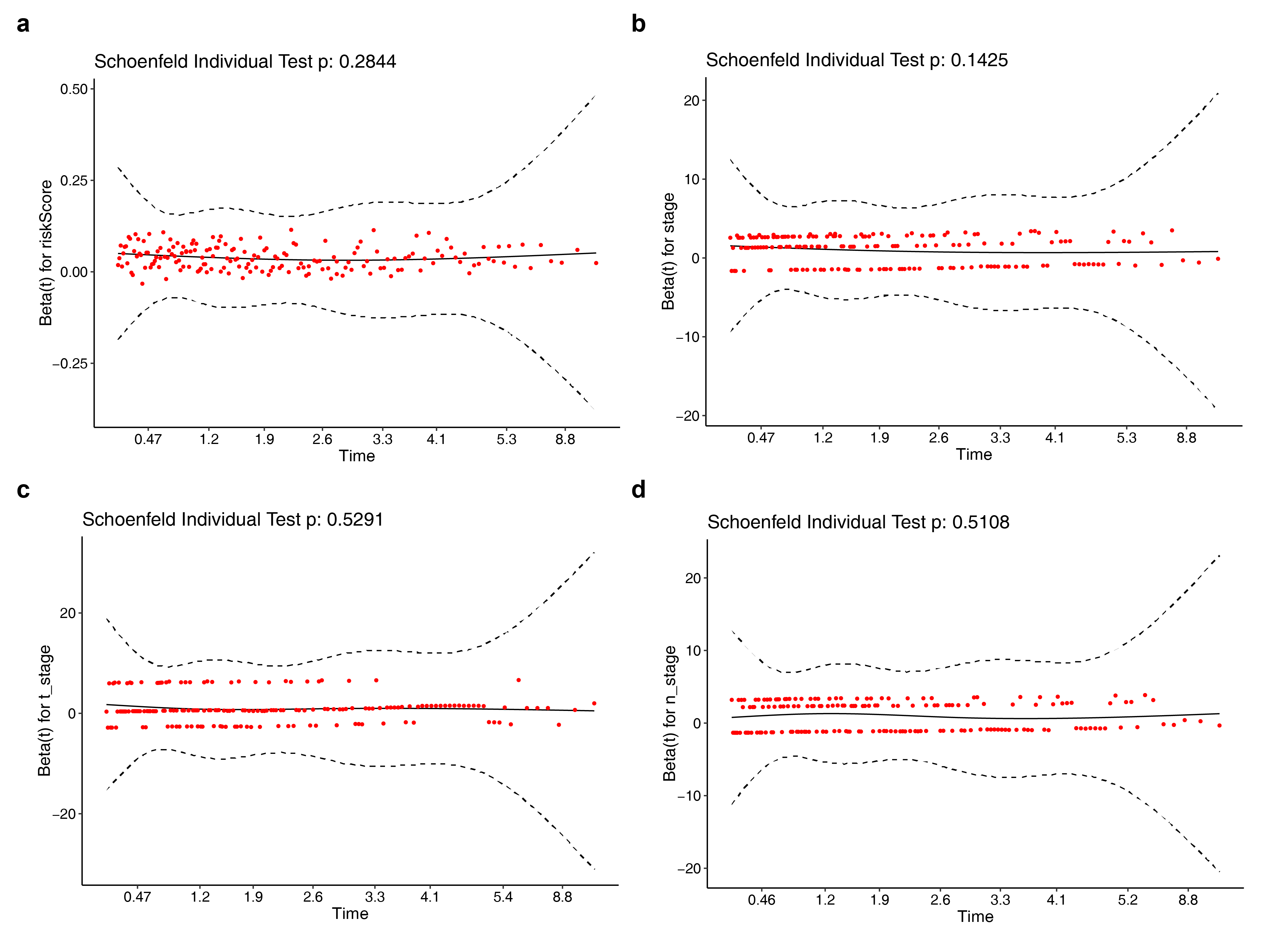

Supplement: Supplementary file 1 [file ijms-27-03065-s001.zip › Figure S3.tif]

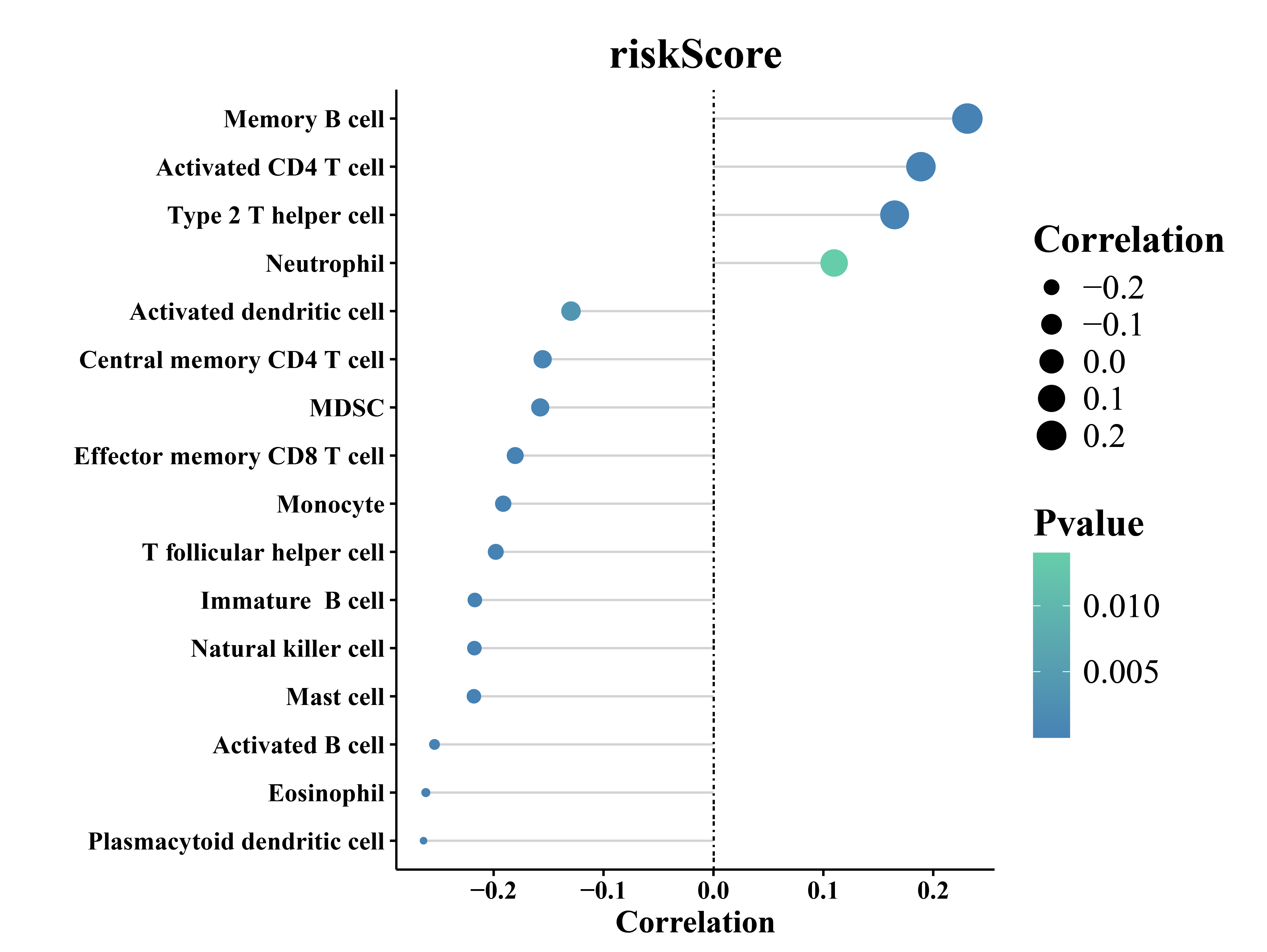

Supplement: Supplementary file 1 [file ijms-27-03065-s001.zip › Figure S4.tif]

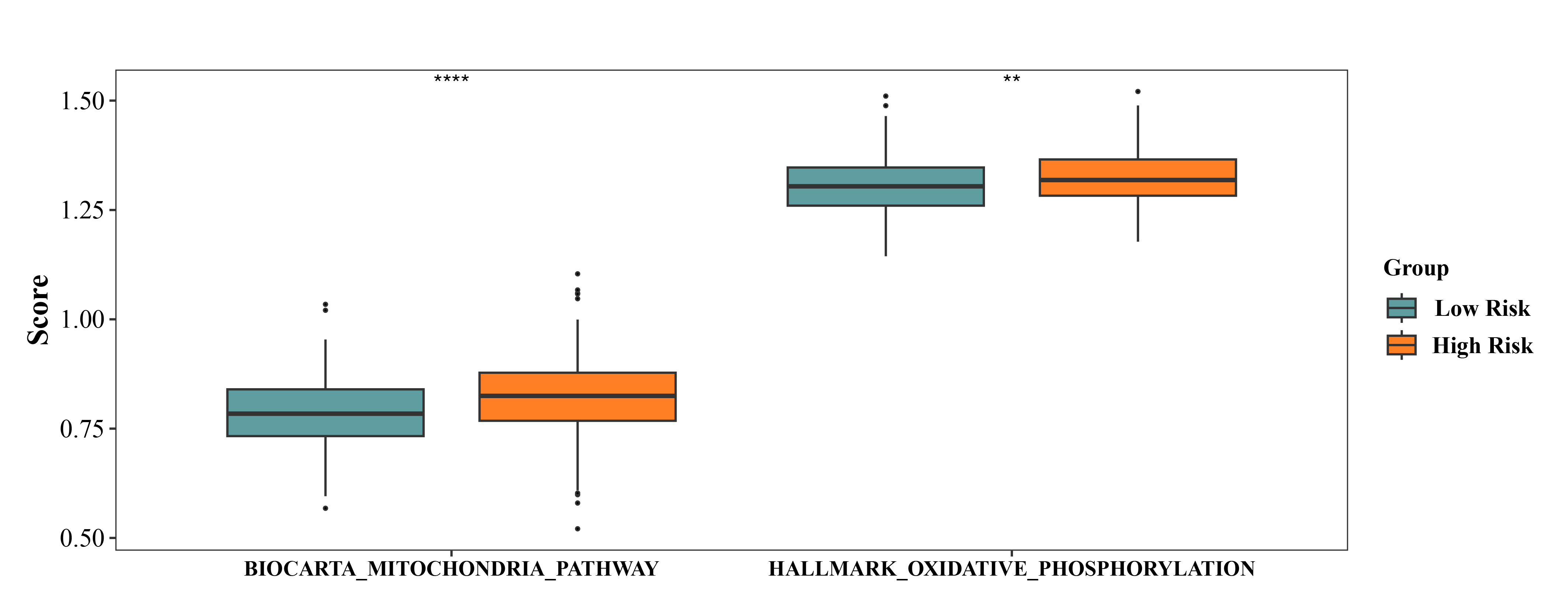

Supplement: Supplementary file 1 [file ijms-27-03065-s001.zip › Figure S5.tif]

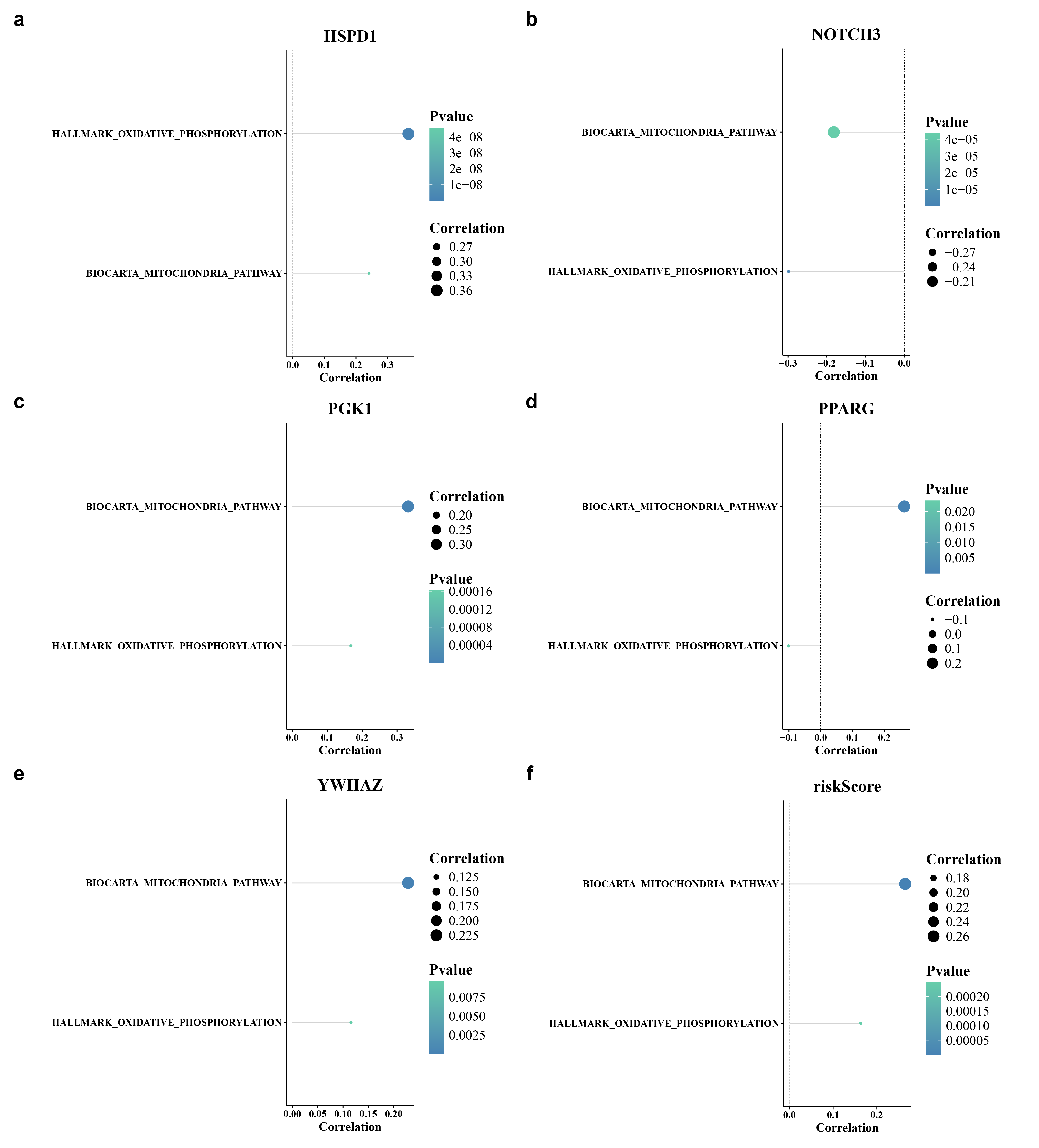

Supplement: Supplementary file 1 [file ijms-27-03065-s001.zip › Figure S6.tif]
